# Mapping Evidence of Impacts of COVID-19 Outbreak on Sexual and Reproductive Health: A Scoping Review

**DOI:** 10.3390/healthcare9040436

**Published:** 2021-04-08

**Authors:** Obasanjo Afolabi Bolarinwa, Bright Opoku Ahinkorah, Abdul-Aziz Seidu, Edward Kwabena Ameyaw, Balsam Qubais Saeed, John Elvis Hagan, Ugochinyere Ijeoma Nwagbara

**Affiliations:** 1 Department of Public Health Medicine, School of Nursing and Public Health, University of KwaZulu-Natal, Durban 4041, South Africa; 219098880@stu.ukzn.ac.za (O.A.B.); microugo4real@yahoo.com (U.I.N.); 2 School of Public Health, University of Technology Sydney, Sydney, NSW 2007, Australia; brightahinkorah@gmail.com (B.O.A.); edmeyaw19@gmail.com (E.K.A.); 3 Department of Population and Health, University of Cape Coast, Cape Coast PMB TF0494, Ghana; abdul-aziz.seidu@stu.ucc.edu.gh; 4 College of Public Health, Medical and Veterinary Services, James Cook University, Townsville, QLD 4811, Australia; 5 L & E Research Consult, Wa 00233, Upper West Region, Ghana; 6 Department of Clinical Sciences, College of Medicine, University of Sharjah, Sharjah 27272, United Arab Emirates; bsaeed@sharjah.ac.ae; 7 Department of Health, Physical Education, and Recreation, University of Cape Coast, Cape Coast PMB TF0494, Ghana; 8 Neurocognition and Action-Biomechanics-Research Group, Faculty of Psychology and Sport Sciences, Bielefeld University, Postfach 10 01 31, 33501 Bielefeld, Germany

**Keywords:** COVID-19, family planning, maternal and child health services, sexual health, sexual and reproductive health

## Abstract

Introduction: The emergence of the coronavirus disease 2019 (COVID-19) pandemic has rapidly transformed the pre-existing worldwide sexual and reproductive health environment. The provision and supply of contraceptives, and a wide variety of sexual health, new-born, and maternal health services have been seriously affected. Thus, this scoping review mapped the available evidence on the impacts of the COVID-19 outbreak on sexual and reproductive health. Methods: Arksey and O’Malley’s methodological framework guided this scoping review. A search was conducted from the following databases: Embase, PubMed, CINAHL, Scopus, WOS, and AJOL. The preferred reporting items for systematic reviews and meta-analyses (PRISMA) chart and PRISMA extension for scoping reviews (PRISMA-ScR) checklist were used to document the review process. The McMaster critical review checklist was used to determine the quality of the included studies. Thematic analyses were conducted using NVivo version 12. Results: Three studies showed evidence on the impact of COVID-19 and family planning services, six studies reported on maternal and child services and eleven studies reported on sexual health (sexual behavior). Limited access to family planning use, reduction in multiple sexual partnership, decreased transactional sex, and maternal and child services disruption were some impacts reported in the included studies. Conclusion: This study has demonstrated the impacts of COVID-19 on family planning access, multiple sexual partnership, transactional sex, and disruption of maternal and child health services. Interventions that will consider the immediate availability of and access to all sexual and reproductive health services should be prioritized.

## 1. Introduction

The emergence of the coronavirus disease 2019 (COVID-19) pandemic has transformed the pre-existing worldwide sexual and reproductive health environment rapidly [1,2]. The provision and supply of contraceptives and a wide variety of sexual health, new-born, and maternal health services have been seriously affected or disrupted [3,4,5].

More than two decades ago, the United Nations at the International Conference on Population and Development (ICPD), held in Cairo, recognized the role of an adequate response to an individual’s reproductive health needs as a panacea to universal economic and social development [6]. The gathering further endorsed the equal distribution of family planning and other related sexual and reproductive health services via the primary healthcare system to encourage comprehensive reproductive health care utilization [6]. Therefore, reproductive health implies an individual’s ability to have a satisfying and safe sex life and have the capacity and freedom to reproduce at their desire [6], hence the need to provide adequate and friendly access to safe, effective, affordable, and acceptable methods of family planning and health care services of their choice that will increase maternal and child health safety. This concept also includes sexual health, which is considered the enhancement of life and personal relations relating to reproduction and sexually transmitted infections (STIs) [6,7].

Prior to the emergence of COVID-19, adverse sexual and reproductive health outcomes have been a major issue globally, with an estimated 210 million women being exposed to pregnancy complications, and approximately half a million of these women dying between pregnancy and the post-partum period [8]. In addition, about 68 000 women die every year from complications of unsafe abortions [9]. In the same vein, 3.3 million infants were stillborn, whilst 3 million babies die in the first week of their life yearly [8]. This is also coupled with more than 340 million new sexually transmitted bacterial and protozoal infections acquired every year [10].

Interestingly, uninterrupted access to sexual and reproductive services has been identified as a remedy to curb these adverse sexual and reproductive health globally and across all socio-demographic characteristics [11,12,13,14]. However, the sudden emergence of the COVID-19 outbreak and the virus’s declaration as a global health concern in the first quarter of the year 2020 led many governments worldwide to enforce country-national lockdowns in order to limit the propagation of the virus [15]. These measures have been documented to have altered sexual and reproductive health (SRH) services around the world [16].

Specifically, SRH services such as family planning supplies [17], sexual health [18], and maternal and child health services [17,19] have been disrupted. To ensure adherence to guidelines prescribed by the World Health Organization (WHO) [20], authors at the Guttmacher Institute and others have opined that the sudden halt in supply of SRH services would lead to an unexpected rise in adverse SRH [17,21,22]. Thus, this study intends to map the evidential impacts of COVID-19 on SRH using a systematic scoping review.

## 2. Materials and Method

### 2.1. Study Design

We performed a scoping review by searching for original articles on the impact of COVID-19 on sexual and reproductive health, which includes family planning services, maternal and child health service, and sexual behavior globally between December 2019 and October 2020 using Arksey and O’Malley’s [23] scoping review framework and Levac et al. [24] methodological enhancement for scoping review projects guidelines. We also followed the preferred reporting items for systematic reviews and meta-analyses extension for scoping reviews (PRISMA-ScR) guidelines [25], while the Joanna Briggs Institute checklist [26], the McMaster critical review checklist [27], and the authority, accuracy, coverage, objectivity, date, and significance (AACODS) checklist [28] were used for the assessment of the studies.

The study protocol was registered at the open science framework (OSF) and can be accessed via https://osf.io/9mz6s.

### 2.2. Research Question

The research question was: what are the impacts of the COVID-19 on sexual and reproductive health in terms of access to family planning, maternal and child health services, and sexual behavior?

In order to effectively answer the research question, we adopted the population, concept, and context (PCC) framework developed by the Joanna Briggs Institute [26] to determine the eligibility of our primary research question, as illustrated in Table 1.

### 2.3. Data sources and Literature Search

We conducted a systematic literature search on studies published in peer-reviewed journals and grey literature with a focus on the study’s research question. Eight electronic databases were searched, and these were Excerpta Medica dataBASE (Embase), PubMed, Cumulative Index of Nursing and Allied Health Literature (CINAHL), Scopus, Web of Science (WOS), and Africa Journals Online (AJOL). A search was also conducted on the Google Scholar website. They were searched for relevant studies published in English between December 2019 and October 2020 with the following key terms: “Coronavirus 2019” OR “COVID-19” with “Family planning use” “Family planning service” OR “Contraceptive use” “Contraception use” “Maternal health service” “Child health service” and “Sexual behavior”.

Boolean terms (AND/OR) were used to separate our keywords. Medical subject headings (MeSH) were also used in the electronic database search. We also searched thoroughly through the reference lists of the included articles to source relevant literature.

### 2.4. Study Selection

We conducted the study selection in three stages. Firstly, we performed a comprehensive title screening from the resources retrieved from the databases mentioned above. Secondly, O.A.B. and B.O.A. excluded studies that did not address the study’s research question along with all the duplicates. Lastly, all included studies that qualified for abstract and full-text screening were uploaded on Endnote X9 software (Clarivate Analytics, Philadelphia, PA, USA) and screened by two reviewers (A.S. and E.K.A.). Discrepancies between the reviewers at abstract and full-text screening stages were resolved by involving a third screener (A.O.B.) through a discussion.

### 2.5. Eligibility Criteria

#### 2.5.1. Inclusion Criteria

The study included qualitative and quantitative peer-reviewed primary studies conducted globally. These include:

- Research articles reporting information regarding the impact of COVID-19 on family planning services, maternal and child health service, and sexual behavior.
- Articles that explored any study design published in peer-reviewed journals addressing the research question.
- Articles published in English.
- Articles published between December 2019 and October 2020.

#### 2.5.2. Exclusion Criteria

Articles such as commentaries and editorials were not considered. The review did not include studies that do not report the impact of COVID-19 on family planning, maternal and child health services, and sexual behavior. The review also excluded studies conducted in any language other than English.

### 2.6. Condition or Domain Being Studied

This review investigated the impact of coronavirus 2019 on sexual and reproductive health globally.

### 2.7. Participants/Population

The population of interest was all persons who have been affected by COVID-19 whilst accessing any sexual and reproductive health service. Additionally, persons who had experienced any setback in sexual and reproductive health as a result of COVID-19 were included.

### 2.8. Data Charting

The included articles in this study were thoroughly read, and data from these articles were organized into different themes using NVivo 10 (QSR International, Burlington, VT, USA) [29]. The data were extracted with the following headings: author and year, study setting (country), study design, population, mean/age range of participants, percentage of males, percentage of females, family planning service and COVID-19, maternal and child health service and COVID-19, sexual behavior and COVID-19.

### 2.9. Quality Assessment of the Included Studies

The quality assessment was conducted by two external, independent reviewers and was verified by O.A.B. and E.K.A. This was done with the McMaster critical review [27].

### 2.10. Collating, Summarizing, and Reporting the Results

A narrative account of the data extracted from the included studies was analyzed using thematic content analysis. Data were extracted around the following outcomes: impact of COVID-19 on family planning services, impact COVID-19 on maternal and health services, and impact of COVID-19 on sexual behavior.

## 3. Results

### 3.1. Screening Results

**Figure 1:**
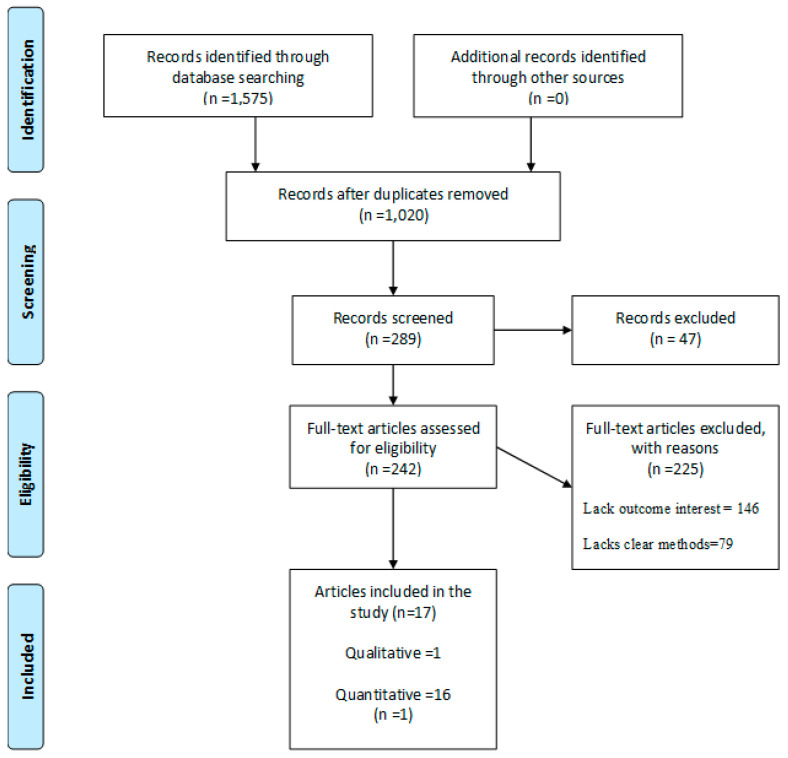
PRISMA flow-chart of the study selection process. (Source: Adapted from Moher et al. [30]).

After the database search, this scoping review found two hundred and eighty-nine (289) eligible studies from a total of 1575 articles after title screening and removing duplicates. A total of 47 articles were also excluded following the abstract and 225 after full article screening. Hence, 17 articles were included for analysis. Results of the article screening are presented in Figure 1.

The preferred report items for systematic and meta-analysis (PRISMA) flow chart for the screening and selection of studies in this review is shown in Figure 1.

### 3.2. Characteristics of the Included Studies

**Figure 2:**
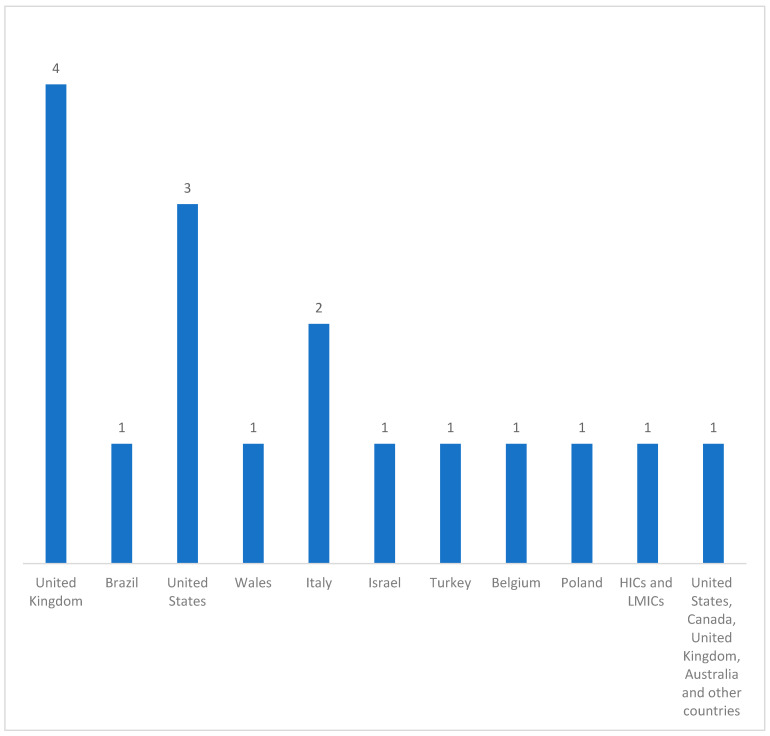
Distribution of the countries represented in the included studies (n = 17).

Table 1 shows the characteristics of the included studies. These included studies were conducted in various high income countries (HICs) and low-middle income countries (LMICs) (Figure 2): 23% of the studies were done in the United Kingdom [31,32,33,34], 6% in Brazil [35], 17% in the United States [36,37,38], 6% in Wales [39], 12% in Italy [40,41], 6% in Israel [42], 6% in the United States, Canada, United Kingdom, Australia, and other countries [43], 6% in Turkey [44], 6% in Belgium [45], 6% in Poland [46], and 6% in HICs and LMICs [47].

Three studies showed evidence on the impact of COVID-19 and family planning services [36,40,44], six studies reported on COVID-19 impact on maternal and child services [31,33,34,44,45,47], and eleven studies reported on COVID-19 and sexual behavior [32,35,36,37,38,39,41,42,43,44,46].

### 3.3. Quality of Evidence from the Included Studies

All the included studies received a high quality score ranging from 80% to 100% during the methodological quality assessment. Overall, the included studies were deemed to have a low chance of bias.

Table 2 below shows that the most common sexual and reproductive health service impacted by the outbreak of COVID-19 was sexual behavior because changes in pattern of sexual behavior were reported in almost all the countries included in the study except in Belgium, with 11 studies reporting unusual sexual behavior during the COVID-19 pandemic [32,35,36,37,38,39,41,42,43,44,46]. Contraceptive use or family planning service disruption was reported in the United States [36], Italy [40], and Turkey [44], with 3 studies reporting limited access to contraceptive of choice. Maternal and child health services disruption was reported in the United Kingdom thrice [31,33,34], Turkey once [44], Belgium once [45], and once in a study conducted in more than one country [47].

### 3.4. Themes from Included Studies

#### 3.4.1. COVID-19 and Family Planning Services

Four of the seventeen included studies reported on the impact of COVID-19 and family planning services [36,37,40,44]. A study conducted in Turkey showed a significant decrease in contraception use during the pandemic compared with the time prior to the pandemic [44].

A cross-sectional study carried out in Italy reported that 50.5% of single or non-cohabiting women had discontinued their short-acting reversible contraception (SARC) method while social distancing due to the COVID-19 pandemic, for non-method-related reasons [40]. However, 46.5% of the non-cohabiting or single women did not adhere to the social distancing guidelines and continued with their sexual activity resulting in 14.9% of the women having an unplanned pregnancy and requesting termination [40]. A study conducted on the impact of COVID-19 on men who have sex with men (MSM) in the United States demonstrated that 9.4% of the participants had less access to condoms, while 5.4% reported less use of a condom [36].

On the contrary, Sanchez et al. [36] reported that 89.4% and 92.9% of the participants had no change in access to or condom use, consecutively, and condom access and usage remained unchanged due to COVID-19 [36] as shown in Table 3.

#### 3.4.2. COVID-19 and Maternal and Child Services

Six of the included studies reported on the impact of COVID-19 and maternal and child services [31,33,34,44,45,47]. A study conducted in the United Kingdom during COVID-19 lockdown showed that 62% of pregnant women felt a lack of interpersonal care while using virtual consultations, which affected how much information they disclosed to their healthcare workers [31]. It further reported that 14% of the participants could not express themselves well over the phone, as they believed that discussing sensitive matters over the telephone was inappropriate [31]. Six percent of the pregnant women had experienced a stillbirth prior to the COVID-19 pandemic and thus would have preferred to have a face-to-face consultation with the health worker to reduce anxiety [31]. Due to the fear of contracting COVID-19, 12% of the participants couldn’t attend routine scan appointments [31]. This finding is similar to a study conducted in Belgium, where 53% of the pregnant women indicated that the coronavirus pandemic influenced their current pregnancy follow-up to some extent [45].

A study conducted in Turkey showed 32.7% of participants planned to become pregnant before the pandemic, but the number decreased significantly to 5.1% throughout the pandemic [44]. A study by McDonald et al. [33] on the impact of COVID-19 on childhood vaccinations in England demonstrated a general decrease in hexavalent vaccinations delivered in 2020 compared with 2019 [33]. Measles-mumps-rubella (MMR) vaccination counts fell from February 2020 before the physical distancing measures implemented in response to the COVID-19 epidemic were introduced. In the first 3 weeks of physical distancing, MMR vaccination counts were 19.8% lower (95% CI: −20.7 to −18.9) than for the same period in 2019 after introducing physical distancing measures [33]. Forty-three percent (43%) and thirty-nine percent (39%) of the breastfeeding women reported having experienced some impact of the pandemic on the extent of medical counselling and social support during the breastfeeding period, respectively [45]. More than 90% of the breastfeeding women refuted that the pandemic affected their breastfeeding practices, nor did they indicate that the coronavirus was responsible for breastfeeding cessation [45]. A study by Saso et al. [47] showed that 50% or more reported issues with either maternal or infant/toddler vaccine delivery within their country [47]. The lockdown measures reduced access for pregnant women and infants from easily attending antenatal clinics and primary health care centers, respectively [47]. Adjustments to clinics, shortage of staff, lack of personal protective equipment (PPE), and vaccine supply problems were the provider issues reported by most participants [47]. A study conducted in the UK assessing the impact of the COVID-19 pandemic on the diagnosis and management of pediatric inflammatory bowel disease (IBD) showed that over 50% of children and young people presenting with a suspected diagnosis of IBD were diagnosed without a histological diagnosis due to restrictions placed on endoscopy at over 90% of centers across the UK [34] as shown in Table 3 above.

#### 3.4.3. COVID-19 and Sexual Behavior

Eleven studies reported the impact of COVID-19 and sexual behavior [32,35,36,37,38,39,41,42,43,44,46] as shown in Table 3 above. Torres et al. (35) conducted a study on the impact of the COVID-19 pandemic on sexual minority populations in Brazil showing that almost half of participants, 45.4%, abstained from sex during the social distancing period [35]. The majority of participants reported a decreased number of sexual partners during the social distancing period, with more than three quarters (76.8%) of respondents mainly finding casual partners online [35]. The study also showed that 28.8% of participants had virtual sex [35]. A study on the impact of COVID-19 on men who have sex with men across the United States showed that 68% of participants had fewer opportunities to have sex with a partner due to COVID-19 [36]. Access to human immunodeficiency virus (HIV) and sexually transmitted infections (STI) testing or treatment centers decreased (25.4%) as a result of the pandemic [36]. Sanchez et al. [36] revealed that 54.9% of participants had trouble getting a pre-exposure prophylaxis (PrEP) prescription while 54.1% also had trouble getting PrEP medication [36]. Another study by Stephenson et al. [37] reported that COVID-19 prevented a small percentage (9%) of the men who have sex with men from accessing their PrEP prescriptions [37].

Gillespie et al. [39] revealed that 42% of participants reported condom-less sex in the period prior to the introduction of social distancing measures and 20% reported condom-less sex afterward [39].

Few participants (1.4%) reported that they had participated in transactional sex for the first time during the COVID-19 lockdown [37]. A study conducted in Israel showed that only 3.2% of the men who have sex with men could imagine themselves having sex with a partner who is infected with COVID-19 compared with 30.1% in the case of HIV [42]. This is so because they perceive the threat of severe acute respiratory syndrome coronavirus to be greater than that of HIV [42]. During the COVID-19 social-distancing period, 39.5% of the participants had met a new casual sex partner while most of them, 84%, had up to 3 sexual partners, and 2.1% met more than 10 sexual partners [42]. A study conducted in Italy on the sexual function and quality of life in reproductive-age women during the social restriction period due to the COVID-19 epidemic revealed that the mean number of sexual intercourses/month decreased significantly from 6.3 ± 1.9 to 2.3 ± 1.8, and 9% women did not have sexual intercourse during the month of social restriction [41]. The number of women who practiced sexual intercourse ≥ 4/month, 4 weeks after the introduction of the social distancing measures due to the COVID-19 outbreak, decreased from 100% to 58.4% [41], while women who had sexual intercourses ≥ 8/month decreased from 34.8% to 9% [41]. Jacob et al. [32] revealed that 40% of the population reported engaging in sexual activity at least once per week on average, hence were classified as sexually active [32].

The occurrence of sexual activity significantly increased from 33.5% in people who were self-isolated for 0–5 days to 47.0% in those who were self-isolated for ≥ 11 days [32]. Starks et al. [38] showed that the unweighted probability of reporting condom-less anal sex (CAS) with a casual partner declined significantly from 71.6% pre-COVID to 26.4% during COVID [38]. A study on changes in sexual behavior during the COVID-19 pandemic revealed that 43.5% of participants reported a decline in the quality of their sex life [43]. A small number of participants (20.8%) reported masturbating once per day or more during the past year. 23.2% reported this frequency since the pandemic began [43]. A study conducted in Turkey on the effect of the COVID-19 pandemic on female sexual behavior showed that the weekly frequency of sexual intercourse was significantly increased during the pandemic compared with the 6–12 months prior [44]. Fuchs et al. [46] revealed that female sexual function index (FSFI) scores of domains such as desire, arousal, lubrication, orgasm, satisfaction, and pain decreased during the pandemic among Polish women [46]. This finding is similar to another study in Turkey that reported that the three domain scores for arousal, orgasm, and satisfaction also decreased during the pandemic [44]. The number of women with sexual dysfunction (overall FSFI score 26 or below) before the pandemic was 15.3% and increased to 34.3% during total lockdown [46].

## 4. Discussion

This review aimed at harnessing evidence on the impact of COVID-19 on sexual and reproductive health. Thus, we mapped the available evidence using a scoping review. The results of our study showed variations in the impact of COVID-19 on sexual and reproductive health globally. Specifically, the review showed that COVID-19 impacted family planning services, sexual behavior, and maternal and child health services.

Our findings on the impact of COVID-19 on family planning services showed discontinuation in family planning’s preferred method because of social distancing [40]. The choice of family planning method has always been a sexual and reproductive health issue prior to the pandemic. However, this impact was felt greatly during the COVID-19 pandemic as a result of limited access to preferred choice [40,48].

The study result showed that despite unchanged access to condoms during COVID-19, participants had fewer sexual partners [36]. This change in behavior could be attributed to COVID-19 social distancing guidelines and stay-at-home orders issued by the national government [49,50]. On the other hand, some studies in this review reported limited access to PrEP prescription and medications [36], less transactional sex, especially among MSM [37] during the COVID-19 outbreak. The implications suggest a need for increased attention from medical caregivers for their patients with sexual behaviors that lead to a higher risk of HIV infections in the coming months and years following the reductions in the pandemic social restrictions.

Other study results showed that a study conducted in Turkey revealed that about 32.7% of participants planned to become pregnant before the pandemic, but the number significantly decreased to 5.1% throughout the pandemic, and the rate of contraception use by women significantly also decreased during the pandemic [44]. It could be that the sexual activity of women during the COVID-19 pandemic was largely affected in Turkey, which is in line with a study conducted in Italy that reported less sexual pleasure or desire in women [51].

Lockdown and social distancing guidelines reduced access for pregnant women and infants from easily reaching antenatal clinics and primary health care centers from receiving vaccines and immunizations [47]. There were some differences between participants from low-middle income countries (LMICs) compared to high-income countries (HICs). The barriers identified in LMICs were mainly provider issues, such as cancelled clinic appointments and unavailability of vaccines. In contrast, participants from HICs reported changes in the clinic format, such as setting up telephone or virtual consultations, rather than indefinite cancellation or suspension of services [47].

Despite the social-distancing regulations and the COVID-19 pandemic lockdown, our study reported that casual sex was common among men who were generally younger, single, and less educated [42]. Prior to the COVID-19 pandemic, unmarried persons, younger, and less educated persons were often reported to engage in casual sex, and this behavior has persisted during the COVID-19 pandemic [52,53,54].

## 5. Strengths and Limitations

An important strength of this study was the exhaustive search for relevant studies for inclusion using different databases. The results of this scoping review followed the PRISMA guidelines, which ensured complete and transparent reporting of this scoping review. The scoping review methodology permitted the inclusion of various study designs and used a systematic approach to identifying relevant studies, charting, and analyzing the outcomes. Despite the strengths mentioned earlier, the following limitations should not be overlooked. This scoping review limited the language of published articles to include only English. This introduced a selection bias and limited retrieval of relevant studies published in other languages.

## 6. Conclusions

This study has demonstrated the impacts of COVID-19 on family planning access, multiple sexual partnerships, transactional sex, and disruption of maternal and child health services. Interventions that will consider all sexual and reproductive health services’ immediate availability should be given priority globally.

## Figures and Tables

**Table 1:** Population concept context (PCC) framework for defining the eligibility of the studies for the primary research question.

Criteria	Determinants	Description
P—Population	All population	All people whose sexual and reproductive health has been impacted by COVID-19.
C—Concept	Family planning services	This is determined by family planning service availability to people in any country. The service includes availability and access to contraceptive service of choice during the COVID-19 pandemic.
	Maternal and child health service	This is determined by maternal and child health service availability to people in any country. The service includes availability and access to maternal and child health services of choice during the COVID-19 pandemic.
	Sexual behavior	This is to determine if there has been any change in sexual behavior in terms of engagement in non-use of condom, multiple sexual partners, or transactional sex during the COVID-19 pandemic.
C—Context	All countries	Any country that reported the impact of COVID-19 on sexual and reproductive health between December 2019 and October 2020.

**Table 2:** Showing common findings in all countries included in the study.

	Countries Included in the Study									
COVID-19 and SHR Services	United Kingdom	Brazil	United States	Wales	Italy	Israel	Turkey	Belgium	Poland	Multiple Countries
COVID-19 and family planning services			X *		X *		X *			
COVID-19 and maternal and child services	XX ***						XX *	XX *		XX *
COVID-19 and sexual behavior	XXX *	XXX *	XXX ***	XXX *	XXX *	XXX*	XXX *		XXX *	XXX *

Source: Authors, X represents family planning services were disrupted by COVID-19 outbreak, XX represents maternal and health care services were disrupted by COVID-19 outbreak, XXX represents COVID-19 impacted sexual behavior, * represents number of times study reported any of the SRH by countries.

**Table 3:** Summary characteristics of the included studies.

Author and Year	Study Setting (Country)	Study Design	Population (n) (Sample Size and Target Pop)	COVID-19 and Family Planning Services	COVID-19 and Maternal and Child Services	COVID-19 and Sexual Behavior
Babu Karavadra et al. and 2020 [31]	United Kingdom	Cross-sectional (Web survey)	Women who were pregnant or delivered during COVID-19, n = 1451		- 62% felt lack of interpersonal care. - 14% cannot express themselves well over the phone. - 2% had mobile issues and could not access maternal healthcare services. - 12% could not attend routine scan appointments due to fears about contracting COVID-19. - 18% felt neglected by health workers.	
Thiago S. Torres et al. and 2020 [35]	Brazil	Cross-sectional (Web-survey)	n = 3486 among cismen			- 24% off-PrEP were at substantial HIV-risk. - 46.6% had access challenge in picking up PrEP medication. - 28.8% had virtual sex. - 10.5% reported condomlessreceptive anal sex with a casual partner.
Travis H. Sanchez et al. and 2020 [36]	United States	Cross-sectional (Web survey)	Men who have sex with men n = 1051	- 9.4% had less access to condoms. - 5.4% reported less use of a condom.		- 68% reported fewer opportunities in having sex with a partner. - 25.4% had less access to HIV/STI testing or treatment centers. - 54.9% having trouble getting PrEP prescription. - 54.1% having trouble getting PrEP medication.
David Gillespie et al. and 2020 [39]	Wales	Longitudinal survey (Web survey)	Participants were individuals accessing PrEP n = 56			- 20% reported condomless sex.
Salvatore Caruso and 2020 [40]	Italy	Cross-sectional (Web survey)	Among women known to be using hormonal contraceptives, n = 169	- 50.5% of non-cohabiting women discontinued SARC use during social distancing, for non-method-related reasons - 14.9% had an unplanned pregnancy.		
Rob Stephenson et al. and 2020 [37]	United States	Cross-sectional (Web survey)	Among gay, bisexual, and other men who have sex with men, n = 518			- 1.4% reported that they had participated in transactional sex. - 9% reported that COVID-19 had prevented them from accessing their PrEP prescriptions.
Guy Shilo and Zohar Mor and 2020 [42]	Israel	Cross-sectional (Web survey)	Among men who have sex with men, n = 2562			- 39.5% met new casual sex partners during this period. - Only 3.2% could imagine themselves having sex with a partner who is infected with COVID-19 compared with 30.1% in case of HIV. - 84% had had up to 3 sexual partners. - 2.1% met more than 10 sexual partners.
Michele Carlo Schiavi and 2020 [41]	Italy	Cross-sectional	Women of reproductive age, n = 89			- 9% of women did not have sexual intercourse during the month of social restriction. - The number of women who practiced sexual intercourses four times/ month, 4 weeks after the introduction of the social distancing measures due to the COVID-19 outbreak, decreased from 89 (100%) to 52 (58.4%). - Women who had sexual intercourse eight times/month decreased from 31 (34.8%) vs. 8 (9%).
Louis Jacob et al. and 2020 [32]	United Kingdom	Cross-sectional (Web survey)	n = 868			- 40% of the population reported engaging in sexual activity at least once per week. - The prevalence of sexual activity significantly increased from 33.5% in people who were self-isolated for 0–5 days to 47.0% in those who were self-isolated for 11 days.
Tyrel J. Starks et al. and 2020 [38]	United States	Cohort-control design	Among sexual minority men, n = 455			- The unweighted probability of reporting CAS with a casual partner declined significantly from 71.6% pre-COVID to 26.4% during COVID.
Justin J. Lehmiller et al. and 2020 [43]	United States 73.4% Canada 6.0% United Kingdom 5.7% Australia 2.4% Other countries, 12.5%	Cross-sectional (Web survey)	Among participants 18 years of age or older, n = 1559			- 43.5% reported a decline in the quality of their sex life. - 20.8% of participants reported masturbating once per day or more during the past year, 23.2% reported this frequency since the pandemic began.
Bahar Yuksel, Faruk Ozgor and 2020 [44]	Turkey	Cross-sectional (Telephone)	Among married patients who were older than 18 years and sexually active, n = 58	- Use of contraception during the pandemic significantly decreased compared with the period before (10 participants vs. 24 participants, *p* = 0.004).	- Before the pandemic, 19 (32.7%) participants intended to become pregnant, however during the pandemic, this number decreased to 3 (5.1%) (*p* = 0.001).	- Average weekly frequency of sexual intercourse was significantly increased during the pandemic compared with the 6–12 months prior (2.4 vs. 1.9; *p* = 0.001).
Helen I McDonald et al. and 2020 [33]	United Kingdom	Cross-sectional	Vaccination among children n = 67,116		- Measles-mumps-rubella vaccination counts fell from February 2020, and in the 3 weeks after the introduction of physical distancing measures were 19.8% lower (95% confidence interval: −20.7 to −18.9) than the same period in 2019. - There was a general decrease in hexavalent vaccinations delivered in 2020 compared with 2019, but no evidence of an increase in the rate of decline with the introduction of physical distancing measures. Counts of both vaccinations increased in weeks 16 and 17, despite physical distancing measures remaining in place.	
Michael Ceulemans et al. and 2020 [45]	Belgium	Cross-sectional (Web survey)	2647 pregnant and 3823 breastfeeding women, n = 6470		- 90% refuted that the pandemic affected their breastfeeding practices, nor indicated that the coronavirus was responsible for breastfeeding cessation. - 86% of all pregnant respondents answered that their pregnancy was mainly followed-up by an obstetrician. - 40% cited that the pandemic negatively influenced the extent of medical counseling by medical specialists. - 43% of the breastfeeding women reported having experienced some impact of the pandemic on the extent of medical counseling during the breastfeeding period. - 39% of the breastfeeding women reported having experienced the impact of the pandemic on the extent of social support they received during the breastfeeding period.	
Anna Fuchs et al. and 2020 [46]	Poland	Cross-sectional	764 sexually active female patients and above 18 years			- Desire, arousal, lubrication, orgasm, satisfaction, and pain decreased. - The number of women with sexual dysfunction (overall FSFI score 26 or below) before the pandemic was 15.3% and increased to 34.3% during the total lockdown.
James John Ashton et al. 2020 [34]	United Kingdom	Cross-sectional	20 tertiaries pediatric IBD centers		- Over 50% of children and young people presenting with a suspected diagnosis of IBD were diagnosed without a histological diagnosis due to restrictions placed on endoscopy at over 90% of centers across the UK. - A total of 122 patients were diagnosed with confirmed, or presumed IBD during April 2020. Of these patients, 53.3% (n = 65) were presumed diagnoses and had not undergone endoscopic or histological examination.	
Anja Saso, Helen Skirrow and Beate Kampmann and 2020 [47]	LMICs and HICs	Cross-sectional (Web survey)	n = 48		- 50% or more reported issues with vaccine delivery within their country. - Lack of access to maternal and child health services. - Provider issues with vaccine supply due to COVID-19.	

## Data Availability

Not applicable.

## Abbreviations

COVID-19	Coronavirus Disease 2019
PRISMA	Preferred Reporting Items for Systematic Reviews and Meta-analyses
PRISMA-ScR	Preferred Reporting Items for Systematic Reviews and Meta-Analyses Extension for Scoping Reviews
ICPD	International Conference on Population and Development
STI	Sexually Transmitted Infections
AACODS	Authority, Accuracy, Coverage, Objectivity, Date, and Significance
OSF	Open Science Framework
PCC	Population, Concept, and Context
Embase	Excerpta Medica database
CINAHL	Cumulative Index of Nursing and Allied Health Literature
WOS	Web of Science
AJOL	Africa Journals Online
SARC	Short-Acting Reversible Contraception
MSM	Men who have sex with Men
MMR	Measles-mumps-rubella
IBD	Inflammatory Bowel Disease
PPE	Personal Protective Equipment
HIV	Human Immunodeficiency Virus
CAS	Condom-less Anal Sex
FSFI	Female Sexual; Function Index
LMICs	Low-Middle-Income Countries
HICs	High Income Countries; PrEP: Pre-Exposure Prophylaxis
